# Supplementary material for: Chromothripsis during telomere crisis is independent of NHEJ, and consistent with a replicative origin
Source: Genome Res. 2019 May;29(5):737–49. doi: 10.1101/gr.240705.118 (PMC6499312; doi:10.1101/gr.240705.118)
Supplement: Supplemental Material [file supp_gr.240705.118_Supplemental_file_1.zip › contigs/annotated_contigs/DB70/contig.4.DB70_length_638_mean_cov_9.09090909091.docx]

**DB70_length_638_mean_cov_9.09090909091**

```
GTGATCGCAGCTCACTGAAACCTCAAAA|TCCAGGGCTCAAGTCATCCCCCCACCTCAGCCTCCTAAGTAGCTGGGACT|GCAGGCATG
 >chr1:77583983-77584011 + E=4e-02  >chr10:67017248-67017298 - E=2e-12          >chr1:2
CACCATTACACCCAGCTACGTTTTGTATTTTTCATAGAGACA|TGAAAAAC|GGCTCACTGCAACCTCCATCTCCCAGGTTCAAGCAAT
38923055-238923106 - E=6e-07 p=0e+00               >chr9:133388891-133389395 + E=2e-257
TCTTGTGCCTCAGCCACCTGAGTAGCTGGGATTACAAGCACACACCACCATGCCCGGCTAATTTTTGTATTTTTAGCAGAGACAGGTTT

TGCCATATTGCCCAGGATGGTCTCAAACTCCTGGCCTCAAATGATCCACCAGCCTCAGCCTCCCAAAGTGCTGGGATTACAGGTGTGAG

CCACCATGCCTGGCCCAGAAAAAATTTTTTATTTCTGATTCCAACATCTTTATCAAAATGTTTTGGTTAATATTCAGATGCATTTCCTG

CCAGTCATTCTTTCATATGCACATTCGTTTACAGGCATGTCAGGTAGTCTGTTTACATTGTGTACCTGTGCATTTGGTTTTTTGTTTTT

CGGGTTTTTTTTTTGAGACAGAGTCTCGCTCTGTCACCCAGGCTGGGGTGCAGCAGCGCGATCTCGGCTCACTGCAACCTACACCTCCC

AGGTTCAAGCGATTCTCCC
```
